# Tranexamic acid did not attenuate the acute rise in plasma syndecan-1 in a severely injured cohort: a laboratory analysis of the PATCH clinical trial

**DOI:** 10.1186/s40635-025-00784-2

**Published:** 2025-07-11

**Authors:** Elissa M. Milford, Dusan Marjanovic, Heidi Ho, Steven Wallis, Dominik F. Draxler, Biswadev Mitra, Russell L. Gruen, Robert Medcalf, Stephen Bernard, Colin McArthur, Marc Meagele, Brian Burns, Dashiell Gantner, Michael C. Reade

**Affiliations:** 1 Intensive Care Services, Royal Brisbane and Women’s Hospital, Butterfield St Herston, Brisbane, QLD 4006 Australia; 2 The University of Queensland, Brisbane, Australia; 3 Monash University, Melbourne, Australia; 4 The Alfred Hospital, Melbourne, Australia; 5 The Australian National University, Canberra, Australia; 6 Auckland City Hospital, Auckland, New Zealand; 7 Medical Research Institute of New Zealand, Wellington, New Zealand; 8 Cologne-Merheim Medical Center, Cologne, Germany; 9 Institute for Research in Operative Medicine, Cologne, Germany; 10 University Witten-Herdecke, Cologne, Germany; 11 Royal North Shore Hospital, Sydney, Australia; 12 Sydney University, Sydney, Australia

**Keywords:** Trauma, Tranexamic acid, Glycocalyx, Endothelium, Syndecan-1

## Abstract

**Background:**

Injury to the vascular endothelium occurs in up to 34% of patients acutely following severe traumatic injury and can be quantified clinically by measuring the plasma concentration of syndecan-1 (SDC-1). Tranexamic acid (TXA) attenuates endothelial damage in animal and cell culture models and has been associated with lower SDC-1 levels after prehospital TXA administration. The aim of this study was to assess the association of prehospital TXA on SDC-1 levels in a more severely injured prehospital cohort.

**Methods:**

The PATCH-Trauma trial randomised patients to receive pre-hospital TXA or placebo. In this sub-cohort, SDC-1 was measured in blood samples collected on hospital admission, at 8 and 24 h. Relationships between SDC-1 levels over time, treatment groups, and outcomes were analyzed using regression modelling controlling for potential confounding factors.

**Results:**

There were 89 patients included, with 57 administered TXA and 32 administered placebo (per protocol). SDC-1 levels were available in 87 patients on arrival to hospital, 70 at 8 h, and 69 at 24 h. Patients had a median SDC-1 on admission of 106 ng/mL (IQR 88–137). There was no effect of TXA treatment on SDC-1 levels over the first 24 h of hospital admission, even after controlling for known confounders. There was no association between SDC-1 level at any time point and the development of deep vein thrombosis or sepsis, mortality at 28-days, or days alive and out of hospital, even after adjustment for confounding factors.

**Conclusion:**

Administration of TXA, initiated pre-hospital, did not affect SDC-1 levels in the first 24 h of hospital admission in this severely injured cohort. Further research is required to elucidate the mechanisms of the effect of TXA on the endothelium as well as the utility of SDC-1 as an endothelial biomarker.

## Background

The vascular endothelium lines the luminal aspect of all blood vessels in the body and consists of a single cell layer of endothelial cells, their basal lamina, and the endothelial glycocalyx [1]. The glycocalyx is a fragile, complex, and dynamic structure composed of proteoglycans and glycoproteins that lines the luminal aspect of the endothelial cells [1]. The glycocalyx has several functions including regulating vascular permeability, coagulation, and inflammation, that when damaged may lead to adverse systemic effects [1]. Systemic endothelial damage and glycocalyx shedding occur early, as soon as within 15 min, in around a third of patients following major traumatic injury and can be quantified by measuring the plasma concentration of breakdown products of the glycocalyx [2, 3]. The main structural backbone of the glycocalyx, a transmembrane bound proteoglycan called syndecan-1 (SDC-1), has become the most frequently measured biomarker for trauma-induced endothelial damage [4]. Shedding of SDC-1 into the circulation occurs from a variety of stimuli, including hypoperfusion, inflammatory mediators, hypoxia, and catecholamines. These lead to cleavage of SDC-1, and other glycocalyx components, from the underlying endothelial cells by proteases including matrix metalloproteases and the ADAM (a disintegrin and metalloproteinase) family of proteases, via a complex and poorly understood web of intermediary pathways [5].

Numerous studies have reported an association between SDC-1 levels on hospital arrival following severe trauma and poor outcomes including increased mortality, hospital length of stay, transfusion volumes, coagulopathy, sepsis, and organ dysfunction [3, 6, 7]. A threshold level of 40 ng/mL has been proposed as the definition of the “Endotheliopathy of Trauma” (EoT) as this level maximized the sensitivity and specificity of mortality prediction in a large trauma cohort [2]. However, this threshold has not been validated in other populations so it not yet considered an established definition of EoT. In addition, it is still unclear whether glycocalyx shedding is a cause or a marker of poor outcomes, and whether repairing the glycocalyx and the underlying endothelium could lead to improved patient outcomes.

While no therapy is yet approved to treat the EoT specifically, several therapies currently used for other indications have attenuated SDC-1 shedding and endothelial hyperpermeability in preclinical studies. These include corticosteroids, doxycycline, fresh frozen plasma and tranexamic acid (TXA) [5, 8–10]. The latter two are frequently used in the trauma population and it is possible that some of their beneficial effects may be due to attenuating endothelial damage.

TXA is a synthetic derivative of the amino acid lysine, and its most well understood effect is the inhibition of plasmin-mediated fibrinolysis. When administered within three hours following major trauma, TXA has been shown to decrease the risk of early mortality [11, 12]. However, the mechanism for this is not well understood, and it is likely that the reduction in bleeding by inhibiting fibrinolysis is only partially responsible. It may have a benefit even when hyperfibrinolysis is not present, and administration beyond three hours post injury has been associated with harm for unknown reasons [11–13].

In endothelial cell culture and animal models, TXA attenuates trauma-induced SDC-1 shedding as well as endothelial, lung, and gut wall hyperpermeability [9, 14–18] via inhibition of glycocalyx-cleaving proteases [16]. However, these endothelial protective effects were not observed when TXA was administered after 120 min in these studies. It is therefore possible that the benefit of TXA following trauma may, at least in part, be attributed to its attenuation of the EoT [9, 16, 17].

A small number of clinical studies have measured endothelial biomarker changes following TXA administration in severe trauma, with mixed results. The largest of these was a post-hoc laboratory analysis of the STAAMP trial, a 927-patient randomised placebo-controlled trial of prehospital administration of TXA following major trauma [8]. A small decrease in SDC-1 concentration at admission and 12 h post admission to hospital was observed in patients treated with prehospital TXA compared to placebo, and a dose effect was also observed [8]. However, the mean SDC-1 level in the STAAMP cohort on arrival to hospital was 30.9 ng/mL, which is below the previously proposed level that defines the presence of EoT (40 ng/mL). It is possible that greater reductions in SDC-1 occur in a more severely injured cohort with higher SDC-1 levels, and there may be heterogeneity of treatment effect in which those with a greater degree of EoT are more likely to benefit from TXA administration.

The Prehospital Administration of Tranexamic Acid in Major Trauma (PATCH-Trauma) trial similarly assessed the effects of prehospital TXA on patient outcomes following trauma [12]. As the inclusion criteria restricted enrolment to patients at high risk of trauma induced coagulopathy, we hypothesized that the PATCH-Trauma cohort would have higher baseline SDC-1 levels than the STAAMP cohort, reflecting a greater degree of EoT, with a corresponding greater reduction in SDC-1 levels following TXA administration. Given the promising preclinical effects of TXA on endothelial integrity and the conflicting clinical evidence, we aimed to investigate the impact of TXA on SDC-1 levels in a severely injured cohort from the PATCH-Trauma trial.

## Methods

### Study design and population

A secondary laboratory analysis in a sub-cohort of the PATCH-Trauma trial was undertaken. The PATCH-Trauma trial (NCT02187120) was a prospective, international, double-blind, randomized, placebo-controlled trial. Patients were eligible for inclusion if they were over 18 years of age, were assessed as being at high risk for acute traumatic coagulopathy using the Coagulopathy of Severe Trauma (COAST) score [19], and could be given the first dose of the study intervention within 3 h of injury and before hospital admission.

A total of 1310 patients were recruited across 15 international sites in the PATCH-Trauma trial. Of these, 661 were randomised to receive TXA, delivered as a 1 g bolus of prehospital TXA followed by a 1 g 8-h infusion on arrival to hospital, and 646 were randomised to receive a double-blinded placebo at the same timepoints. The likelihood of survival with a favorable outcome at 6 months was not increased by TXA in this trial (53.7% in the TXA group vs 53.5% in the placebo group (risk ratio, 1.00; 95% CI, 0.90 to 1.12; P = 0.95). There were no differences in serious adverse events between the two groups [12].

The sub-cohort for this laboratory analysis consisted of a convenience sample, dependent on the availability of research staff to collect samples, of a sub-cohort of patients from three participating centres (Royal Brisbane and Women’s Hospital, Queensland, Australia; Gold Coast University Hospital, Queensland, Australia; The Alfred Hospital, Victoria, Australia).

### Blood sample collection and endothelial biomarker analysis

Blood samples were collected at time of arrival to hospital (baseline), and 8 and 24 h after admission to hospital. Blood was collected into lithium heparin tubes and centrifuged at 3000 × g for 10 min at room temperature immediately after collection. The plasma fractions of the samples were stored at −80 °C and batch processed at the conclusion of the trial. SDC-1 was measured as a biomarker of EoT at all three time points in the plasma samples using a commercially available enzyme linked immunosorbent assay according to the manufacturer’s instructions (Diaclone, France) within 6 years of sample collection. No samples had undergone a freeze–thaw cycle prior to SDC-1 measurement.

### Statistical analysis

Differences in SDC-1 levels and outcomes were compared between treatment groups by per-protocol using the Wilcoxon Rank Sum test for continuous variables (as nearly all variables were not normally distributed) and the χ^2^ test for categorical variables. All patients received either 0 g, 1 g, or 2 g of TXA during the trial intervention period (when accounting for protocol deviations independent of treatment group assignment). Given there were only 14 patients that received 1 g (the prehospital dose only), and it is biologically plausible that the prehospital TXA dose has the greatest impact on SDC-1 shedding, the cohort were dichotomized into placebo (0 g) and TXA (1 or 2 g) per-protocol groups.

The lme4 package (1.1–35.5) in RStudio (version 2024.09.0 + 375) was used to perform a linear mixed effects analysis of the relationship between SDC-1 levels and per-protocol treatment group over the first 24 h post-hospital admission. Fixed effects were treatment group and timepoint (including the interaction term). Injury Severity Score (ISS), Glasgow Coma Scale (GCS), FFP units within 24 h of injury, and admission lactate were included as covariates in a separate multivariable model. These covariates were prospectively chosen as known confounders in this population (that is, are associated with an elevated SDC-1, expected to be associated with the outcomes and not in the causal pathways) [2, 6, 20, 21]. Random effects were a random intercept for each individual to allow for the likely correlated SDC-1 values within individuals over time. *P*-values were obtained by likelihood ratio tests of the model with the effect in question against the model without the effect in question. Overly influential participants were detected and the effect of their removal from the mixed-effects models were assessed by calculating the Cook’s distance and standardized difference of the beta (DFBETAS) using cutoffs of 4/n and 2/√n respectively, using the influence.ME package in R [22].

As days alive and out of hospital at day 90 (DAOH_90_) had a bimodal distribution and did not meet the assumptions for standard linear regression analysis, linear quantile regression using a null hypothesis of equality in the median was used to assess the effect of SDC-1 at all three timepoints on DAOH_90_ using the quantreg package (version 5.99.1) in RStudio (version 2024.09.0 + 375).

Continuous variables are presented as medians with interquartile range (IQR), and categorical variables are presented number and percentages. Statistical significance was determined at the probability (*p*) < 0.05 level. Analyses were performed using RStudio version 2024.09.0 + 375.

## Results

Blood samples were analyzed for SDC-1 concentration in 87 patients on arrival to ED (56 in the per-protocol TXA group, 31 in the per-protocol placebo group), 70 at 8 h (42 in the TXA group, 28 in the placebo group), and 69 at 24 h (43 in the TXA group, 26 in the placebo group). There were 62 patients who had a blood sample analyzed at all three time points (38 in the TXA group, 24 in the placebo group). There were no patients who died before the 8-h sample could be collected, and 3 patients who died before the 24-h sample could be collected. The additional missing samples were due to research staff unavailability to collect samples.

The baseline variables were similar between the two per-protocol treatment groups, apart from GCS, with a greater proportion of patients with a GCS less than 9 in the TXA group, and a lower proportion with a GCS 13–15, compared to the placebo group (44% vs 22%, and 47% vs 75% respectively; *p* = 0.04) (Table 1). The imbalance between treatment groups in terms of GCS category was not present in the overall trial cohort [12]. The median volume of crystalloid administered in the first 4 h of hospital admission was greater in the TXA group (1696 mL, IQR 1148–2916 vs 3000 mL, IQR 1582–4148; *p* = 0.032).

**Table 1:** Baseline characteristics and physiology by per-protocol treatment group in a sub-cohort of the PATCH trial

	All patients (n = 89)	Placebo (n = 32)	TXA (n = 57)	*p*
Baseline characteristics and physiology				
Sex—Male	67 (75%)	22 (69%)	45 (79%)	0.416
Age (years)	40 (27–57)	40 (28–56)	40 (27–57)	0.617
ISS	34 (22–48)	29 (18–46)	38 (22–48)	0.653
Injury type				0.653
… Blast	1 (1%)	0 (0%)	1 (2%)	
… Blunt	79 (89%)	28 (88%)	51 (89%)	
… Penetrating	9 (10%)	4 (12%)	5 (9%)	
PH SBP (mmHg)	80 (70–90)	80 (66–88)	80 (70–90)	0.384
PH GCS				0.04*
… < 9	32 (36%)	7 (22%)	25 (44%)	
… 9–12	6 (7%)	1 (3%)	5 (9%)	
… 13–15	51 (57%)	24 (75%)	27 (47%)	
Time between injury and hospital arrival (minutes)	120 (96–150)	120 (102–144)	126 (90–168)	0.723
Time between injury and first study drug dose (minutes)	85 (65–123)	82 (66–115)	92 (62–130)	0.572
Admission SBP (mmHg)	118 (95–138)	119 (100–136)	116 (92–138)	0.869
Admission INR	1.3 (1.1–1.5)	1.2 (1.1–1.4)	1.4 (1.2–1.6)	0.051
Admission lactate (mmol/L)	3.1 (1.8–4.3)	2.4 (1.6–3.8)	3.3 (1.8–5.1)	0.146
Resuscitation fluids and blood products				
PH RBC (units)	4 (2.8–4)	4 (3.5–5)	4 (2–4)	0.55
PH crystalloid volume (mL)	2200 (1938–2750)	2100 (1625–2200)	2500 (2125–3625)	0.198
RBC in first 4 h post admission (units)	4 (2–7)	4.5 (4–7)	4 (2–6)	0.382
FFP in first 4 h post admission (units)	3 (2–5)	3 (2–4)	3.5 (2–6)	0.764
Crystalloid volume in first 4 h post admission (mL)	2252 (1410–3697)	1696 (1148–2916)	3000 (1582–4148)	0.032*
RBC between 4 and 24 h post admission (units)	2 (2–3)	2 (1–3.5)	2 (2–3)	0.752
FFP between 4 and 24 h post admission (units)	2 (1.2–3)	2 (1.2–3.5)	2 (1.8–3)	1
Crystalloid volume between 4 and 24 h post admission (mL)	1222 (610–2167)	1000 (580–1524)	1697 (657–2492)	0.104

Values are reported as median (IQR) and counts (percentages)

^*^ < 0.05

ISS, injury severity score; PH, pre-hospital; SBP, systolic blood pressure; GCS, Glasgow Coma Scale; INR, international normalized ratio; SDC-1, syndecan-1; RBC, red blood cells; FFP, fresh frozen plasma

### TXA did not reduce SDC-1 plasma levels in this cohort

**Fig. 1:**
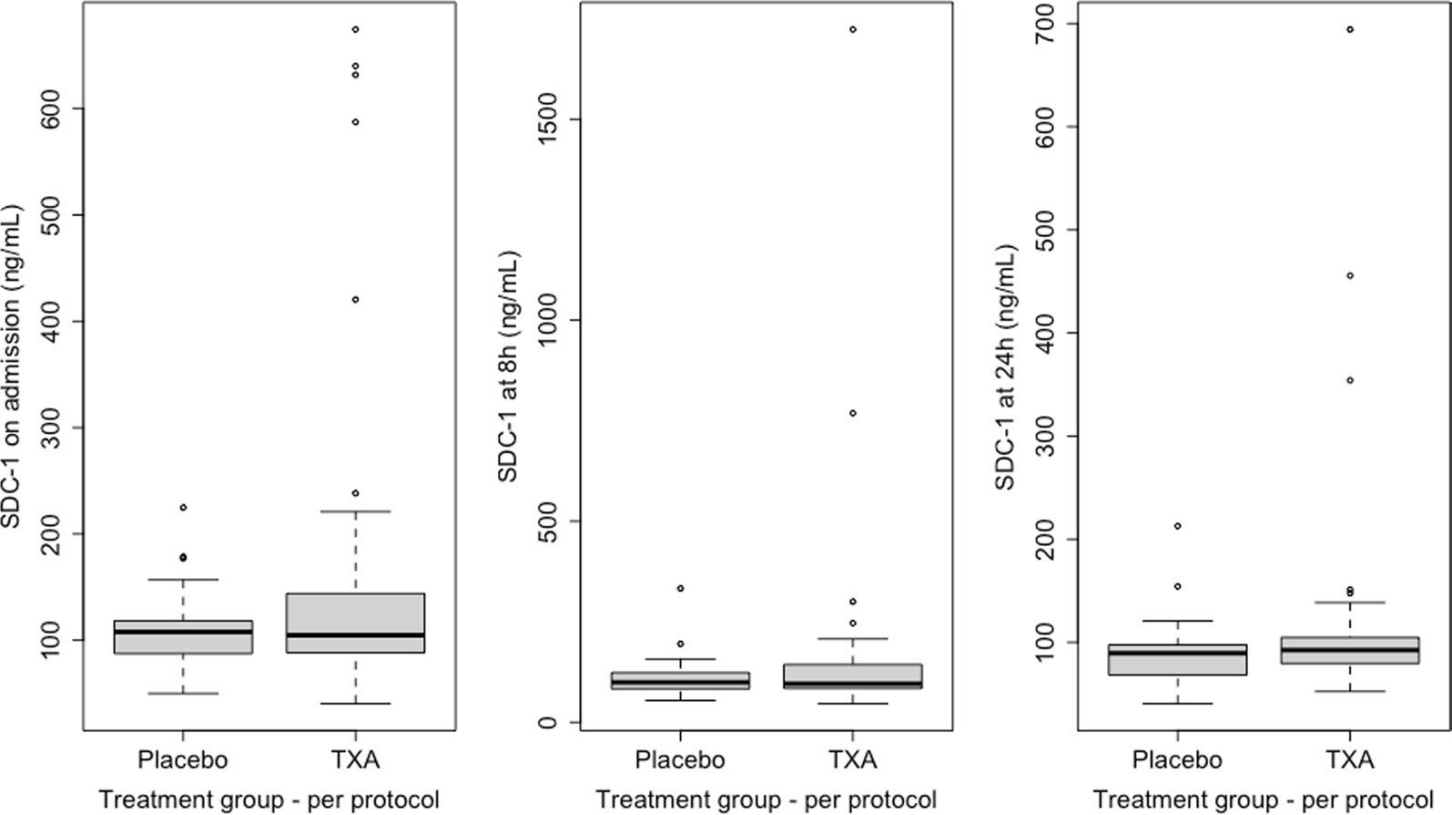
Median (IQR) syndecan-1 (SDC-1) levels on admission, 8h, and 24h post-admission in the per-protocol placebo and tranexamic acid (TXA) groups

Patients had a median SDC-1 on admission of 106 ng/mL (IQR 88—137), and all patients had a SDC-1 level greater than the proposed definition of EoT of 40 ng/mL. There was no difference in SDC-1 levels on arrival to hospital, or at 8 h or 24 h after arrival to hospital between the per-protocol placebo and TXA groups (Fig. 1 and Table 2). Using log transformed values of SDC-1 in a linear mixed effects model, there was no effect of TXA treatment on SDC-1 levels over the first 24 h of hospital admission (estimate for effect of TXA treatment: 1.22 ng/mL increase in SDC-1 over 24 h, 95% CI 0.97 to 1.52; *p* = 0.59), even after adjusting for injury severity, prehospital GCS, FFP administered in first 24 h, crystalloid administered in first 4 h post admission, and admission lactate (estimate 1.72 ng/mL increase in first 24 h, 95% CI 1.14 to 2.59; *p* = 0.40).

**Table 2:** Syndecan-1 levels on admission, 8 h, and 24 h, and outcomes by per-protocol treatment group

	All patients (n = 89)	Placebo (n = 32)	TXA (n = 57)	*p*
SDC-1				
SDC-1 on admission (ng/mL)	106 (88–137)	108 (87–118)	105 (89–144)	0.449
SDC-1 8 h (ng/mL)	97 (84–128)	99 (83–121)	96 (84–139)	0.876
SDC-1 24 h (ng/mL)	91 (79–101)	90 (70–97)	93 (80–105)	0.359
Outcomes				
Any episode of sepsis	27 (30%)	9 (28%)	18 (32%)	0.92
Time between admission and first sepsis diagnosis	5 (4–6)	4 (2–5)	6 (4–7)	0.072
Any DVT detected	25 (28%)	8 (25%)	17 (30%)	0.81
Time between admission and first DVT diagnosis	5 (4–8)	5 (3.8–6)	5 (4–8)	0.68
Died within 28 days	10 (11%)	1 (3%)	9 (16%)	0.143
Time between injury and death	1 (0–7.8)	18 (12–24)	1 (0–1)	0.148
Cause of death^#^				0.134
… Exsanguination	1 (1%)	0 (0%)	1 (2%)	
… Head injury	10 (11%)	1 (3%)	9 (16%)	
… Multi-organ failure	1 (1%)	1 (3%)	0 (0%)	
DAOH_90_	67 (51–78)	74 (58–82)	66 (47–78)	0.081

Values are reported as median (IQR) and numbers (percentages)

^#^ More than one cause of death was possible

ISS, injury severity score; DAOH_90_, days alive and out of hospital to day 90

There were several outlier SDC-1 levels at all timepoints as illustrated in Fig. 1. There were 10/87, 8/70, and 4/69 patients with SDC-1 levels greater than 1.5 IQRs above the 0.75 quantile at admission, 8 h, and 24 h respectively. The influence of outliers on the mixed effects models was assessed by re-evaluating the models following the exclusion of overly influential participants from the dataset. The conclusions of the univariable and multivariable models did not change with the exclusion of these overly influential participants.

### SDC-1 plasma levels were not associated with days alive and out of hospital

There was also no association between SDC-1 levels at any time point and DAOH_90_ in an unadjusted and adjusted (adjusted for ISS, age, treatment group, and prehospital GCS) linear quantile regression model at the 0.5 quantile (Table 3). Repeating the analysis with removal of outliers at each time point (defined as 1.5 IQRs above the 0.75 quantile) did not change the conclusions of the regression models for any of the assessed outcomes.

**Table 3:** Linear quantile regression at the 0.5 quantile of effect of SDC-1 on DAOH_90_

	Univariable		Multivariable	
	Estimate (95% CI)	*p*	Estimate (95% CI)	*p*
SDC-1 on admission	− 0.095 (− 0.128 to 0.006)	0.072	− 0.02 (− 0.06 to 0.02)	0.537
SDC-1 8 h	− 0.044 (− 0.145 to 0.045)	0.141	− 0.02 (− 0.03 to 0.07)	0.392
SDC-1 24 h	− 0.06 (− 0.12 to 0.01)	0.311	− 0.05 (− 0.08 to 0.02)	0.260

Covariates in multivariable analysis are injury severity score, age (years), and prehospital Glasgow Coma Scale

SDC-1, syndecan-1 (ng/mL); DAOH_90_, days alive and out of hospital to day 90

## Discussion

In this post-hoc secondary analysis of an endothelial biomarker in a sub-cohort of 89 severely injured patients from the PATCH-Trauma clinical trial, all patients had a SDC-1 level greater than the proposed (but not yet widely validated) definition of EoT of 40 ng/mL. However, we did not observe an association between the administration of prehospital TXA and lower plasma levels of SDC-1 in the first 24 h following admission to hospital. We also did not find an association between SDC-1 and the risk of death within 28 days of injury, the development of sepsis or a DVT, or DAOH_90_.

It is likely that this study was underpowered to detect a difference in SDC-1 levels due to the small size of the subset of patients with available blood samples, and because other studies have suggested the effect size is small. There have only been three other studies that have assessed the effect of TXA on SDC-1 levels in a human clinical cohort. The largest of these was a post hoc laboratory analysis of 766 patients from the Study of Tranexamic Acid During Air and Ground Prehospital Transport (STAAMP) Trial [8]. In this trial, trauma patients were randomized to receive 1 g of prehospital TXA, followed by either placebo, 1 g, or 2 g of TXA on arrival to hospital, with all doses finished by 8 h of hospital admission [23]. Blood samples were collected at time of hospital admission, 12 h, 24 h, and 72 h after admission. SDC-1 was an average of 5.2 ng/mL lower on arrival to hospital in the TXA group (33.5, IQR 23–54 ng/mL vs 28.3, IQR 20–43; p = 0.001), a 15% reduction from the placebo group. The STAAMP SDC-1 analysis cohort had a lower median ISS than our cohort (STAAMP cohort: median ISS 11, IQR 4–21 in placebo group vs 12, IQR 5–21 in TXA group; our cohort: median ISS 29, IQR 18–46 placebo group vs 38, IQR 22–48 in control group). We hypothesized that because our cohort was more severely injured than the STAAMP cohort, this would result in higher baseline SDC-1 levels and a greater effect from TXA in reducing SDC-1 that we may be able to detect with our smaller sample size. While the baseline SDC-1 levels were higher in our cohort (median of 108 ng/mL, IQR 87–118, in the placebo group on hospital admission) and all patients had a level > 40 ng/mL, the effect of TXA treatment on SDC-1 level did not reach the threshold for statistical significance. However, the differences in point estimates were small with relatively narrow confidence intervals, which suggests a larger sample size may not result in a statistically significant effect. Another possible explanation is that high SDC-1 levels signify a level of physiological derangement that is too severe to benefit from TXA. Future studies could investigate this by measuring baseline SDC-1 levels and assessing for heterogeneity of treatment effect based on baseline SDC-1 level.

A retrospective observational cohort study of a similar number of patients to our study (91 patients) also did not find a difference in SDC-1 concentration between those that did and did not receive TXA [3]. The third study was a randomized clinical trial of prehospital TXA in patients with isolated TBI. This study measured SDC-1 in 285 patients post-study drug administration and found the levels were lower in those that received TXA compared to placebo, and that there was no difference in SDC-1 between early (< 45 min) vs later (> 45 min) administration of TXA [24]. However, the assay used in this study reported SDC-1 levels in the pg/mL range, which is substantially lower than the ng/mL range used in most other studies of EoT, so these results may not be comparable. In any case, future studies of the effect of TXA on the EoT should aim for sufficient power to detect an effect size [8].

It is also possible that we did not observe an effect of TXA on SDC-1 levels due to imbalances in prognostic factors, favoring the placebo group. The TXA group had a greater proportion of patients with a GCS < 9 and a lower proportion with a GCS 13–15 than the placebo group, as well as a greater median volume of crystalloid administered in the first 4 h post admission. In addition, although not statistically significant, the median admission INR and lactate level were higher in the TXA group. Differences in these potentially confounding factors may have contributed to higher SDC-1 levels in the TXA group. There was still no effect of TXA administration on SDC-1 over the first 24 h of admission when the multivariable regression model adjusted for ISS, admission lactate, FFP, crystalloid administration, and prehospital GCS, but this does not account for any unknown unbalanced covariates. Future studies could account for baseline differences in SDC-1 by collecting pre-TXA administration blood samples, however, this proved very challenging logistically in our study, given the nature of the prehospital environment and the multiple concurrent needs of severely injured patients.

The small effect of TXA on the reduction of SDC-1 levels observed in the STAAMP secondary analysis, and the lack of effect observed in our study, could also be due to drug dose. The STAAMP analysis observed a modest dose response relationship of a 4 ng/mL reduction in SDC-1 at 12 h for every 1 g of TXA given over the first 8 h of injury. The TXA concentration shown to attenuate SDC-1 and glycocalyx shedding in several cell culture models was 150 uM, which is approximately equivalent to a 2 g loading dose clinically [9, 16, 17]. Larger doses of TXA may therefore result in a greater attenuation of SDC-1 shedding. Identifying the optimal dose, including consideration of re-dosing with ongoing hemorrhage, that maximizes the net effect of beneficial and adverse effects (such as from increased risk of venous thromboembolic [VTE] complications) warrants further investigation. It is also possible that the practice of administering a second dose over an 8 h period following the first dose may not be beneficial, or even be harmful, given the relationship between time and TXA administration observed in both clinical and preclinical studies.

We did not find an association between SDC-1 level at any timepoint and fewer days alive and out of hospital (a composite measure of mortality and hospital length of stay) in this cohort, even when adjusted for potential confounders. This may also be due to the small sample size of our cohort and baseline imbalances, as several other studies have reported an independent association between SDC-1 level on admission to hospital and poor outcomes [3, 6, 7]. Further work is needed to determine the cause of excess deaths from EoT, noting in our study there was only 1 patient who died from multi-organ failure, 1 patient who died from exsanguination, and the remaining 10 patients died from head injury. We may have observed too few deaths attributable to EoT to have detected a difference.

It is also possible that SDC-1 is not an accurate clinical biomarker for the EoT and for the effects of TXA on the EoT. The sources, metabolism, and clearance of circulating SDC-1 in a heterogenous clinical population are not well understood. In cell culture models and short duration animal studies, SDC-1 levels correlate reasonably with other measurements of endothelial integrity [9, 25]. Clinically, following a severe traumatic injury, SDC-1 can be significantly elevated as soon as within 15 min [3]. It can be rapidly cleared from the circulation when a shedding stimulus resolves (such as in vascular surgery), and it can also remain persistently elevated for as long as a shedding stimulus is ongoing (such as seen in sepsis) [26, 27]. Its metabolism and clearance beyond this are not well understood. Some studies have reported that renal failure has little impact on SDC-1 clearance [28], while others have concluded that it is renally cleared based on plasma and urinary SDC-1 concentration changes. However, SDC-1 is expressed on the renal tubules which confounds the assessment of renal clearance based on urinary SDC-1 concentration [29]. There are also other sources of SDC-1 in the body besides the endothelium such as plasma cells [30], and various chronic diseases such as diabetes are associated with higher baseline levels of SDC-1 [31]. In several studies in trauma, including this one, there are unexplained outliers of SDC-1 levels that are disproportionate to the influence of predictors such as ISS or lactate level. More research is needed to explore the utility of SDC-1 as a biomarker for the EoT in the trauma population.

Another limitation of this study are the differences in the sub cohort and the overall PATCH trial cohort. In particular, the ISS was a median of 34 (IQR 22–48) in the sub cohort compared to 29 (IQR 18–41) in the full trial cohort, although the 28-day mortality was lower in the sub cohort at 11%, compared to 17.3% in the TXA group and 21.8% in the placebo group in the full trial cohort. These differences suggest that the SDC-1 levels measured in the sub cohort may not be representative of the SDC-1 levels of the full trial cohort.

In conclusion, we did not observe an effect of prehospital TXA administration on SDC-1 levels over the first 24 h of hospital admission in a sub-cohort of the PATCH-Trauma clinical trial. Possible reasons for not observing an effect in this study include the small sample size and the consequent risk of a type 2 error, and imbalances in prognostic factors between groups. Further research is required to elucidate the mechanisms of the effect of TXA in trauma including the potential role of the endothelium, the effect of dose and timing of TXA administration on the EoT, as well as the utility of SDC-1 as a biomarker of endothelial damage and repair.

## Data Availability

The datasets used and/or analysed during the current study are available from the corresponding author on reasonable request.
